# Degradation of the Plant Defense Signal Salicylic Acid Protects *Ralstonia solanacearum* from Toxicity and Enhances Virulence on Tobacco

**DOI:** 10.1128/mBio.00656-16

**Published:** 2016-06-21

**Authors:** Tiffany M. Lowe-Power, Jonathan M. Jacobs, Florent Ailloud, Brianna Fochs, Philippe Prior, Caitilyn Allen

**Affiliations:** a Microbiology Doctoral Training Program, University of Wisconsin—Madison, Madison, Wisconsin, USA; b Department of Plant Pathology, University of Wisconsin—Madison, Madison, Wisconsin, USA; c Institut de Recherche pour le Développement, UMR Interactions Plantes Microorganismes Environnement, Montpellier, France; d UMR Peuplements Végétaux et Bioagresseurs en Milieu Tropical, CIRAD-INRA, Saint-Pierre, La Réunion, France; e Laboratoire de la Santé des Végétaux, Agence Nationale Sécurité Sanitaire Alimentaire Nationale, Saint-Pierre, La Réunion, France

## Abstract

Plants use the signaling molecule salicylic acid (SA) to trigger defenses against diverse pathogens, including the bacterial wilt pathogen *Ralstonia solanacearum*. SA can also inhibit microbial growth. Most sequenced strains of the heterogeneous *R. solanacearum* species complex can degrade SA via gentisic acid to pyruvate and fumarate. *R. solanacearum* strain GMI1000 expresses this SA degradation pathway during tomato pathogenesis. Transcriptional analysis revealed that subinhibitory SA levels induced expression of the SA degradation pathway, toxin efflux pumps, and some general stress responses. Interestingly, SA treatment repressed expression of virulence factors, including the type III secretion system, suggesting that this pathogen may suppress virulence functions when stressed. A GMI1000 mutant lacking SA degradation activity was much more susceptible to SA toxicity but retained the wild-type colonization ability and virulence on tomato. This may be because SA is less important than gentisic acid in tomato defense signaling. However, another host, tobacco, responds strongly to SA. To test the hypothesis that SA degradation contributes to virulence on tobacco, we measured the effect of adding this pathway to the tobacco-pathogenic *R. solanacearum* strain K60, which lacks SA degradation genes. Ectopic addition of the GMI1000 SA degradation locus, including adjacent genes encoding two porins and a LysR-type transcriptional regulator, significantly increased the virulence of strain K60 on tobacco. Together, these results suggest that *R. solanacearum* degrades plant SA to protect itself from inhibitory levels of this compound and also to enhance its virulence on plant hosts like tobacco that use SA as a defense signal molecule.

## IMPORTANCE

Plant pathogens such as the bacterial wilt agent *Ralstonia solanacearum* threaten food and economic security by causing significant losses for small- and large-scale growers of tomato, tobacco, banana, potato, and ornamentals. Like most plants, these crop hosts use salicylic acid (SA) both indirectly as a signal to activate defenses and directly as an antimicrobial chemical. We found that SA inhibits growth of *R. solanacearum* and induces a general stress response that includes repression of multiple bacterial wilt virulence factors. The ability to degrade SA reduces the pathogen’s sensitivity to SA toxicity and increases its virulence on tobacco.

## INTRODUCTION

Salicylic acid (SA) is a key signaling molecule for plant defense against certain pathogens (1, 2). As pathogens invade and grow in plant hosts, pathogen activity releases damage-inducing molecular patterns, such as cell wall breakdown products (3, 4). Plants also recognize conserved microbial molecules, such as flagellin, lipopolysaccharide, and chitin, collectively called microbe-associated molecular patterns (5). Host pattern recognition receptors bind these molecular patterns, which initiates a signaling cascade that is amplified by the production of the phenolic defense hormone SA (1). SA activates expression of antimicrobial defense genes such as *PR1*, leading to reinforced cell walls, reduced nutrient availability, and accumulation of antimicrobial chemicals (6, 7). Like many other plant-pathogenic bacteria, the bacterial wilt pathogen *Ralstonia solanacearum* deploys a suite of type III secreted effectors to suppress this pattern-triggered immunity (PTI) and manipulate host biology (8). Certain type III effectors, e.g., RipAA, RipP1, and RipP2, limit the host range of *R. solanacearum* strains, because these effectors are recognized by plant resistance (R) proteins (9, 10). Host recognition of effectors may then activate defense signaling pathways, including the SA pathway, leading to effector-triggered immunity (11). The result of these signals is either quantitative resistance that slows pathogen growth or rapid programmed cell death, known as the hypersensitive response (HR). Plants produce high local concentrations of SA during the HR, which leads to host tissue collapse that deprives pathogens of resources (12). SA also triggers systemic acquired resistance, a form of longer-term immune memory (13). Thus, SA drives bacterium-plant interactions, particularly in the roots, where it restricts many soil bacteria from invading endophytic compartments (14).

The soil-dwelling plant pathogen *R. solanacearum* enters its hosts via root openings and colonizes its preferred niche, the water-transporting xylem vessels (15–18). In the xylem, *R. solanacearum* grows to high cell densities (>10^9^ CFU/g stem) that reduce the flow of xylem sap, resulting in host wilting and death. In late-stage disease, the bacterium exits the host root and infests the soil. *R. solanacearum* strains form a genetically diverse species complex composed of four phylotypes (I to IV) that correspond to evolutionary and geographic origin (19). The *R. solanacearum* species complex as a whole has a host range spanning more than 250 plant species, but no individual strain infects all hosts (20).

Several lines of evidence suggest that SA-mediated defenses protect plant hosts against bacterial wilt disease. Pretreating tomato plants with SA before inoculation with *R. solanacearum* delays the onset and reduces the severity of wilting symptoms (21). During *R. solanacearum* infections, moderately resistant tomato plants strongly express SA-dependent genes like *PR-1a* while they are still asymptomatic, but susceptible tomato plants do not highly express *PR-1a* until wilt symptoms appear (22). Additionally, *R. solanacearum* uses the conserved type III secretion system effector RipR (formerly PopS) to suppress SA-dependent defenses (21). SA has not been directly measured in plants infected by *R. solanacearum*. However, since SA levels increase in a broad diversity of plants responding to microbial infection (23), and we previously showed that expression of genes in the SA defense pathway increases in tomato and tobacco plants infected with *R. solanacearum* (A. Milling and C. Allen, unpublished data) (22), it is reasonable to assume that plants produce SA when infected with *R. solanacearum*.

**FIG 1:**
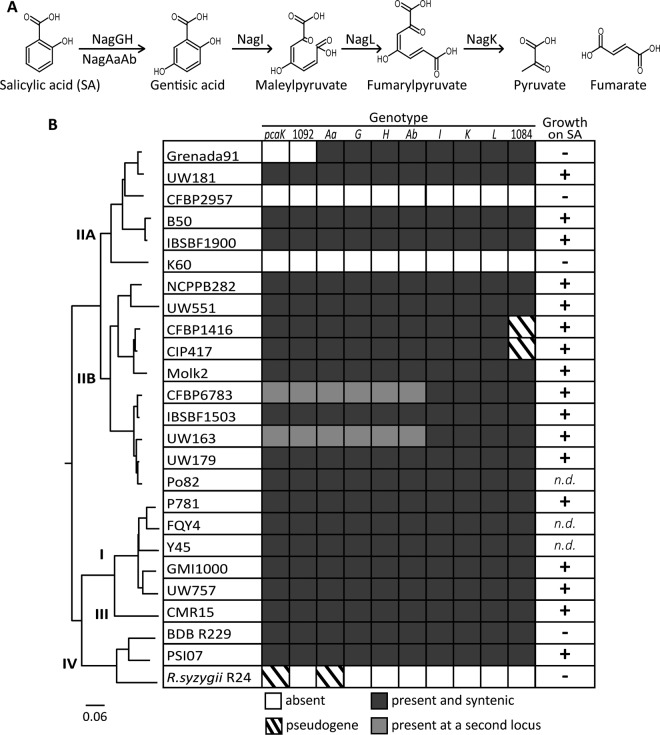
SA degradation is generally conserved in the *R. solanacearum* species complex. (A) The *nag* SA degradation pathway in *R. solanacearum*. (B) Conservation of SA degradation in the *R. solanacearum* species complex. (Left) A whole-genome comparison phylogenetic tree of *R. solanacearum* strains constructed using the maximal unique matches (MUM) index (77). Phylotypes (I to IV) are indicated at the dividing branch points. (Center) Genetic conservation (>80% amino acid identity by BLASTp and synteny with the GMI1000 locus) of *nagAaGHAbIKL* encoding the SA degradation pathway, RSc1092 (1092) encoding a LysR-type transcriptional regulator, and two genes encoding porins, *pcaK* and RSc1084 (1084). Dark gray indicates the gene is present at a single *nag* locus. Light gray indicates that the gene is present but located at a different genomic locus. White indicates that the gene is absent, and diagonal lines indicate the gene is present as a putative pseudogene. (Right) Ability of strains to grow on SA. +, growth was observed on minimal medium plates supplemented with 2 mM SA; −, growth was not observed; n.d., growth was not determined because the authors of the genome announcements would not share the strains.

Intriguingly, *R. solanacearum* may degrade this key plant defense molecule*.* The genome of strain GMI1000 contains the seven-gene *nagAaGHAbIKL* locus predicted to encode the degradation of SA to Krebs cycle intermediates (Fig. 1A) (24–27). These genes are expressed by *R. solanacearum* cells growing in tomato xylem (28, 29). The Nag pathway degrades SA via the phenolic intermediate gentisic acid (Fig. 1A) (26). Gentisic acid is also a defense signaling molecule that accumulates in tomato and cucumber infected with compatible pathogens. Because exogenous SA is converted to gentisic acid in tomato plants (30), gentisic acid signaling likely contributes to the delay in bacterial wilt symptoms observed on tomato plants pretreated with SA (21). However, gentisic acid does not function in defense signaling of all plants; for example, tobacco plants do not convert exogenous SA to gentisic acid (30). We hypothesized that degradation of the defense signaling molecules SA and gentisic acid contributes to *R. solanacearum* virulence. Apart from its function as a defense signal, SA is also a phenolic compound that has direct antimicrobial effects (31–34). Our previous research demonstrated that *R. solanacearum* protects itself from other toxic plant molecules via both efflux pumps and enzymatic degradation (31, 35). It is thus possible that the SA degradation pathway protects this widespread pathogen from an inhibitory chemical.

We investigated the biological role of SA degradation by using a combination of bioinformatic, biochemical, and biological analyses. We show that the SA degradation pathway is conserved in most *R. solanacearum* isolates with sequenced genomes. Transcriptomic profiling and direct inhibition assays indicated that SA is toxic to *R. solanacearum*. Studies with mutants revealed that the bacterium protects itself from SA toxicity with its SA degradation pathway. Moreover, we demonstrate that ectopic expression of the SA degradation genes in *R. solanacearum* strain K60 increases its virulence on tobacco plants.

## RESULTS

### Genetic and functional conservation of SA degradation in the *R. solanacearum* species complex.

We probed the genomes of 25 diverse strains in the *R. solanacearum* species complex for the presence of SA degradation genes to assess the genetic conservation of this trait. Genetic conservation was determined as >80% amino acid similarity with strain GMI1000 proteins by BLASTp analysis and genetic synteny (36). Eighty-eight percent of the strains (22 strains) possessed the *nagAaGHAbIKL* genes, predicted to encode the SA degradation enzymes (Fig. 1B). These genes were absent from two phylotype IIA strains (CFBP2957 and K60) and from the fastidious phylotype IV clove pathogen strain *Ralstonia syzygii* R24 (37, 38). Although the *nag* genes were located in a single cluster in most strains, *nagAaGHAb* and *nagIKL* were in two distant locations in the phylotype IIB strains CFBP6783 and UW163 (39). A putative phenolic-transporting porin gene, *pcaK* (strain GMI1000 locus tag RSc1093) and a LysR-type transcriptional regulator gene (RSc1092) were located upstream of *nagAa* in all *nag*-containing genomes except for that of phylotype IIA strain Grenada91 (39). A second porin (RSc1084) was located downstream of *nagL* in all *nag*-containing genomes, but the gene was predicted to be a pseudogene in the draft genomes of phylotype IIB strains CFBP1416 and CIP417 (39).

We predicted that the strains encoding *nagAaGHAbIKL* would grow on SA as a sole carbon source. Seventy-seven percent (17/22 strains) of the tested strains grew on SA (Fig. 1B). As expected, strains K60, CFBP2957, and *R. syzygii* R24, which lack the *nag* genes, did not grow on SA (Fig. 1B). Surprisingly, phylotype IIA strain Grenada91 did not grow on SA even though its genome apparently encodes the seven *nag* genes. This suggested that an additional factor is required for growth on SA, such as the *pcaK* porin and/or the LysR transcriptional regulator RSc1092, which are both absent from strain Grenada91. CFBP1416 and CIP417 grew on SA despite the predicted pseudogenization of the RSc1084 porin gene, suggesting this gene is either not essential for SA degradation or that sequencing or assembly errors inaccurately predicted pseudogenization. The fastidious phylotype IV Blood Disease Bacterium strain BDB R229 did not grow on SA even though it has the genes encoding the Nag enzymes, PcaK, the LysR regulator, and the RSc1084 porin (Fig. 1B).

### The *nag* operon enables growth on SA and gentisic acid.

**FIG 2:**
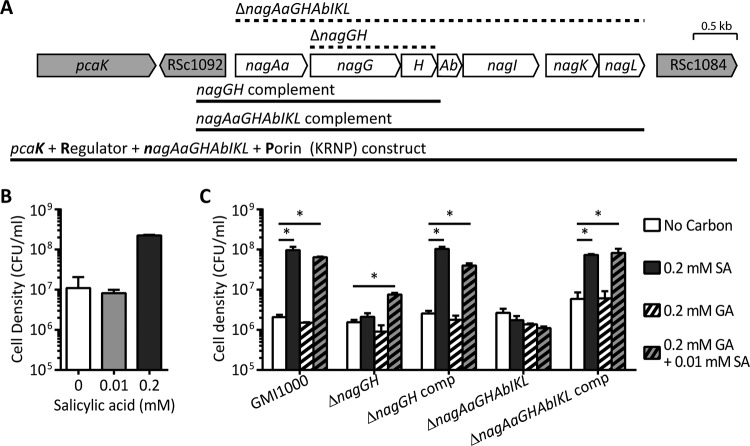
The *nag* genes are required for growth on SA. (A) Structure of the SA degradation gene cluster in *R. solanacearum* strain GMI1000. Regions deleted in the mutants are indicated by dotted lines above the map. The complementation constructs and the region used to introduce SA degradation ability to K60 (KRNP) are indicated by solid lines below the map. (B and C) Growth of GMI1000 *nag* mutants on SA and gentisic acid. Strains were grown at 28°C in liquid minimal medium with the indicated carbon source and concentration. Cell density was measured after 48 h. The averages of three biological replicates are shown, with bars indicating standard errors. Asterisks indicate *P* was <0.05 (Student’s *t* test).

The high conservation of the SA degradation pathway in the *R. solanacearum* species complex suggested that the ability to break down this plant defense signaling molecule contributes to the fitness of this plant pathogen. To measure the contribution of SA degradation to virulence of *R. solanacearum* strain GMI1000, we created two mutants: a Δ*nagGH* mutant that is blocked at the first step of the pathway, and a Δ*nagAaGHAbIKL* mutant that lacks the entire SA degradation pathway (Fig. 2A). As expected, the wild-type strain GMI1000 grew on 0.2 mM SA as a sole carbon source, while the Δ*nagGH* and the Δ*nagAaGHAbIKL* mutants did not (Fig. 2C). We predicted that the wild type and the Δ*nagGH* mutant would grow on the intermediates of the SA degradation pathway, while the Δ*nagAaGHAbIKL* mutant would not. We tested this hypothesis by using the commercially available intermediate, gentisic acid. Surprisingly, none of the strains, including wild-type GMI1000, grew on gentisic acid as a sole carbon source (Fig. 2C). Hypothesizing that expression of the catabolic *nag* genes requires SA as an inducer, strains were grown in 0.2 mM gentisic acid medium supplemented with 10 µM SA. This concentration was not enough SA to serve as a carbon source, since growth of strains in 10 µM SA was indistinguishable from growth in the no-carbon control (Fig. 2B). Exposure to this low SA concentration allowed the wild type and Δ*nagGH* mutant to grow on gentisic acid, but the Δ*nagAaGHAbIKL* mutant lacking the complete pathway still did not grow on gentisic acid (Fig. 2C). Genetic complementation of the Δ*nagGH* and Δ*nagAaGHAbIKL* mutants restored growth on SA and gentisic acid. This result suggests that expression of the *nag* genes is induced by SA but not by the intermediate, gentisic acid.

### Transcriptional response of *R. solanacearum* to SA.

**FIG 3:**
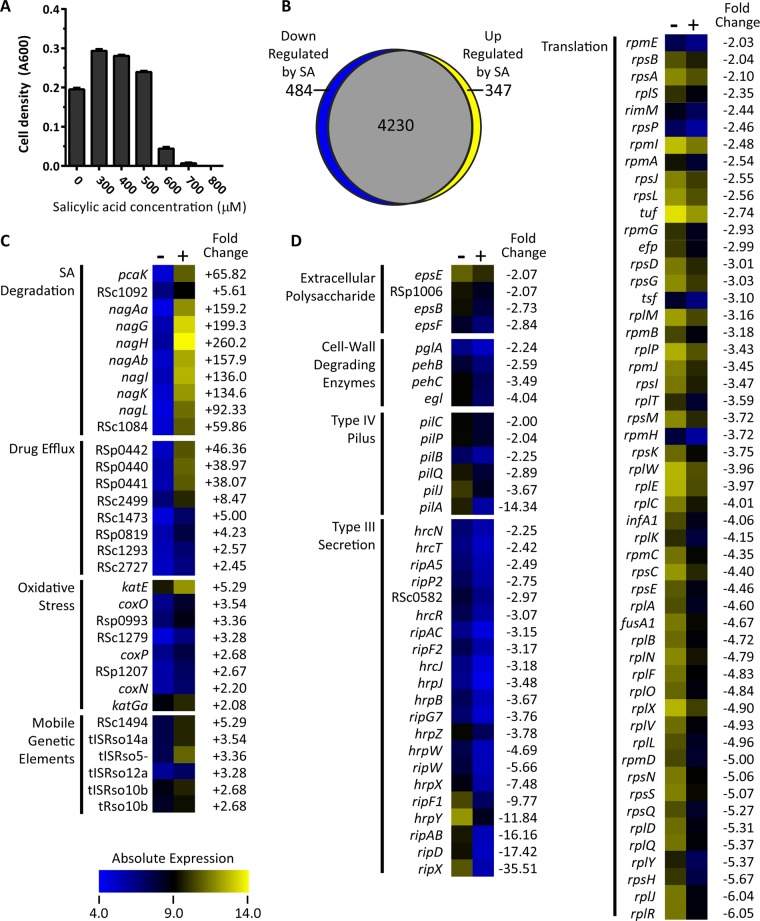
SA modulates *R. solanacearum* gene expression. (A) SA inhibits *R. solanacearum* growth. Strain GMI1000 was grown in minimal medium plus succinate with increasing SA concentration. Cell density was measured based on the *A*_600_ after 48 h. The averages of three biological replicates with standard errors are shown. (B) A proportional Venn diagram of expression patterns created using BioVenn (78). Gene expression was measured on a custom-designed *R. solanacearum* strain GMI1000 microarray chip as previously described (28). ORFs with relative expression levels in SA medium greater than 2-fold different and adjusted *P* values of <0.05 were classified as differentially expressed. (C and D) Heat maps show absolute expression of genes induced (C) and repressed (D) by SA. The gene class is listed to the left of the gene name/gene locus. Heat maps indicating low absolute expression (blue; 4.0) to high absolute expression (yellow; 14.0) are shown to the right of gene names. The fold change (0 µM SA versus 500 µM SA) is shown to the right of each heat map. Heat maps were generated in MeV (version 4.9; Dana-Farber Cancer Institute; http://www.tm4.org/mev.html).

To understand the global response of *R. solanacearum* to SA, we profiled the transcriptomic response of strain GMI1000 to 500 µM SA (Fig. 3). This concentration was chosen because it does not inhibit growth of strain GMI1000 (Fig. 3A) and is in the range of SA concentrations observed in plants responding to pathogens (40). RNA was harvested from cells grown for 3 h in complete minimal medium with or without 500 µM SA. Microarray analysis revealed that 831 genes (16.4% of the CDS represented on the array) were differentially expressed more than 2-fold in response to SA (*P* < 0.05) (Fig. 3B; see also Table S1 in the supplemental material). Predictably, the *nag* genes themselves were most induced by SA, at >92-fold (Fig. 3C). Quantitative reverse transcription-PCR (qRT-PCR) confirmed that both 50 and 500 µM SA induced high expression of the *nag* genes (see Fig. S1 in the supplemental material). SA also induced expression of the porins *pcaK* and RSc1084 by 65.8-fold and 59.9-fold, respectively, and induced expression of the LysR-type regulator RSc1092 by 48.8-fold. This result suggests that RSc1092 positively regulates expression of the *nag* genes and that the PcaK and RSc1084 porins import SA to the cytosol, where the Nag enzymes are predicted to localize based on SignalP 4.1 predictions (41).

Many of the genes that were differentially expressed in the presence of SA encode stress response proteins. Eight genes encoding putative drug efflux pumps were upregulated following SA exposure. The notably high expression levels of putative drug efflux pump genes RSp0440 to RSp0442 (increased 38- to 46-fold by 500 µM SA) suggest that this pump could export SA. Oxidative stress genes, such as *katE* and *coxO*, and genes from mobile genetic elements, such as integrases and phage elements, were also induced by SA.

SA repressed expression of 484 genes (9.5% of the CDS on the array) (Fig. 3D). These included 52 genes encoding translation machinery: ribosomal proteins, translation initiation and elongation factors, *groES* and *groEL*, tRNA synthetases, and tRNA genes.

Interestingly, SA also repressed expression of genes encoding many known bacterial wilt virulence factors. The type III secreted effector *ripX* (formerly *popA*) was the most downregulated gene with known function (35.5-fold-lower expression in 500 µM SA). Moreover, SA repressed an additional 20 genes encoding type III effectors and secretion machinery. SA also repressed *pilC*, *pilP*, *pilB*, *pilQ*, *pilJ*, and *pilA*, which encode components of the type 4 pilus, an appendage used for attachment and twitching motility and that contributes to bacterial wilt virulence (42, 43). Most of the bacterium’s consortium of plant cell wall degradation genes, including pectinase genes *pehA*, *pehB*, and *pehC* and the cellulase *egl*, were repressed by SA (17). Additionally, SA repressed several genes encoding synthesis of the major virulence factor, extracellular polysaccharide (*epsE*, RSp1006, *epsB*, and *epsF*) (22, 44). Because *ripX* was the most SA-responsive virulence factor gene, we investigated whether SA had a dose-response effect on *ripX* expression levels. qRT-PCR confirmed that 500 µM SA repressed *ripX* expression, but 5 and 50 µM SA did not affect *ripX* expression (see Fig. S1 in the supplemental material).

### The Nag pathway protects *R. solanacearum* from toxicity of SA but not of gentisic acid.

**FIG 4:**
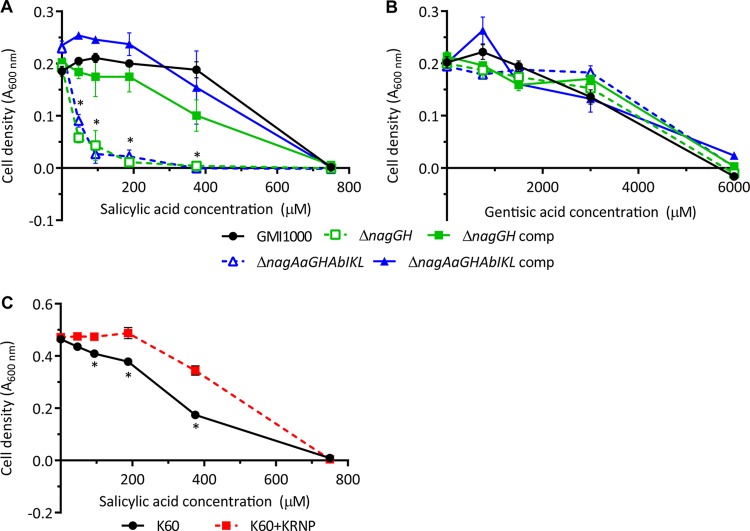
The Nag pathway protects *R. solanacearum* from toxicity of SA, but not from that of gentisic acid. Strains were grown in minimal medium plus succinate with increasing SA (A and C) or increasing gentisic acid supplemented with constant 10 µM SA (B) to induce *nag* gene expression. Cell density was measured based on the *A*_600_ after 48 h. The average results of three biological replicates with standard errors are shown. At time points marked with an asterisk, the SA-degrading strains (GMI1000 or K60+KRNP) grew better than strains that cannot degrade SA (the Δ*nagGH* and Δ*nagAaGHAbIKL* mutants and wild-type K60) (*P* < 0.005; Student’s *t* test).

Although SA is best known as a plant defense signaling molecule, SA is also antimicrobial, like many plant phenolic compounds (31–34). For example, SA toxicity influences the microbial community composition on human skin, where SA is applied in acne face creams, and the rhizosphere, where SA is released in root exudates (14, 45). We hypothesized that the Nag degradation pathway protects *R. solanacearum* from the toxicity of SA and gentisic acid. To determine the toxicity of SA to *R. solanacearum*, we measured growth inhibition in complete minimal medium with increasing concentrations of SA. The MIC of SA for wild-type strain GMI1000 was 600 µM, and the strain grew normally in the presence of 0 to 500 µM SA (Fig. 3A and 4A). In contrast, as little as 47 µM SA inhibited growth of both the Δ*nagGH* and Δ*nagAaGHAbIKL* mutants, demonstrating that the Nag pathway protects *R. solanacearum* from SA toxicity.

Gentisic acid is an understudied plant defense signal that accumulates in tomato and cucumber plants responding to certain bacterial and viral pathogens (30, 46). Because gentisic acid may accumulate during bacterial wilt disease, we tested the ability of the Nag pathway to protect *R. solanacearum* from gentisic acid toxicity. We performed the growth inhibition assay described above with increasing gentisic acid concentrations plus a constant 10 µM SA to induce *nag* gene expression. With an MIC of 6 mM, gentisic acid was 10-fold less toxic than SA to strain GMI1000 (Fig. 4B). The Δ*nagAaGHAbIKL* mutant, which cannot degrade gentisic acid, was as resistant to gentisic acid as the wild-type strain. This suggests that while the Nag pathway intermediate gentisic acid is not highly toxic, the Nag pathway does not protect *R. solanacearum* from it.

### SA degradation contributes to virulence on tobacco plants.

**FIG 5:**
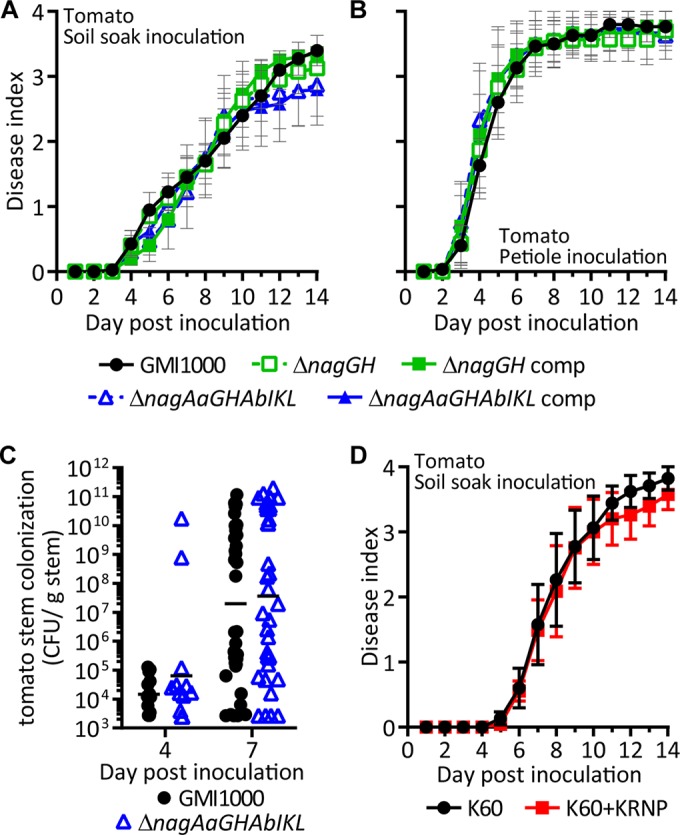
The Nag pathway does not contribute to virulence of *R. solanacearum* on tomato. (A) Seventeen-day-old tomato plants with unwounded roots were soil soak inoculated by pouring bacterial suspensions into the pots (1 × 10^8^ CFU/g soil). Symptoms were rated on a 0 to 4 disease index scale corresponding to the percentage of wilted leaves. (B) Twenty-one-day-old tomato plants were petiole inoculated by placing a suspension of 500 cells on a freshly cut branch. (C) Bacterial density in stem tissue was quantified by dilution plating stem tissue from soil soak inoculated plants. Each symbol represents the population size in a single plant, and horizontal lines represent the geometric means. (D) Seventeen-day-old tomato plants with unwounded roots were soil soak inoculated with SA-degrading strain K60+KRNP or the isogenic strain that does not degrade SA (K60). Virulence of strains (A, B, and D) were not significantly different (*P* > 0.05; repeated measures ANOVA). Bacterial populations in tomato stem (B) were not significantly different (*P* > 0.05 at 4 and 7 days post-soil soak inoculation; Mann-Whitney test).

To determine whether SA degradation contributes to *R. solanacearum* virulence on tomato plants, we inoculated wilt-susceptible tomato plants with wild-type GMI1000 and the Δ*nagGH* and Δ*nagAaGHAbIKL* SA degradation mutants (Fig. 5A and B). Both mutants retained full wild-type virulence on tomato whether the bacteria were applied using a naturalistic soil-soaking inoculation or directly introduced to the xylem via a cut leaf petiole. Additionally, the Δ*nagAaGHAbIKL* mutant colonized tomato stems as well as the wild-type parent strain did following soil soak inoculation (Fig. 5C).

**FIG 6:**
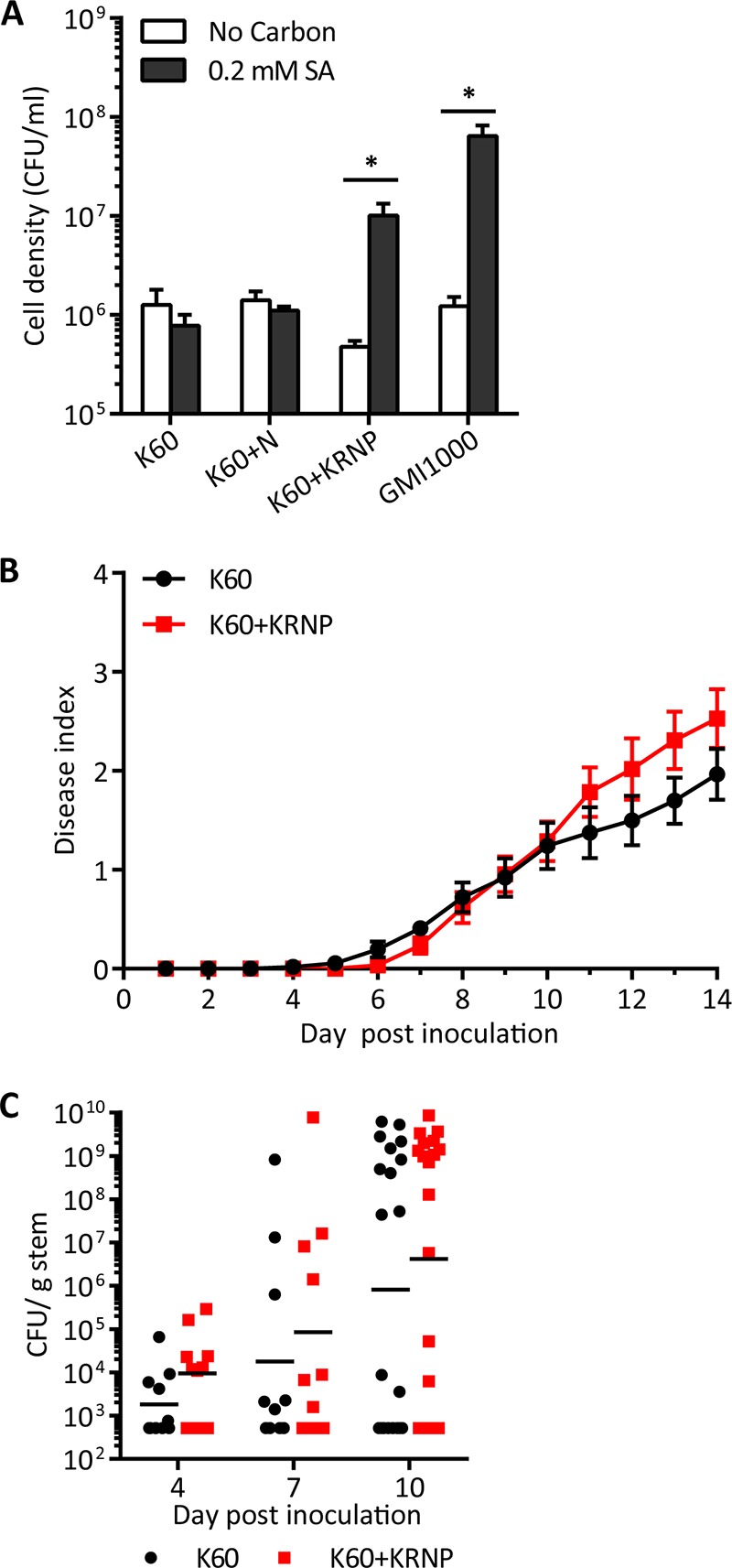
SA degradation increases virulence of *R. solanacearum* strain K60 on tobacco. (A) *In vitro* growth of K60 wild-type (K60), K60 with *nagAaGHAbIKL*_GMI1000_ genes (K60+N), K60 with *pcaK*_GMI1000_, RSc1092_GMI1000_, *nagAaGHAbIKL*_GMI1000_, and RSc1084_GMI1000_ (K60+KRNP), and GMI1000 wild type on SA as sole carbon source. Asterisks indicate *P* was <0.05 (Student’s *t* test). (B) Virulence of strains K60 and K60+KRNP following soil soak inoculation of 3- to 4-week-old tobacco with unwounded roots. Data are the average results for 6 experiments with 45 total plants per strain (*P* < 0.05; repeated measures ANOVA). (C) *R. solanacearum* density in tobacco stem following soil soak inoculation. Each symbol represents the bacterial population size in a single plant (*P* > 0.05 at 4, 7, and 10 days post-soil soak inoculation; Mann-Whitney test).

Tobacco, another *R. solanacearum* host, metabolizes SA differently than tomato plants (30, 46). While tomato plants accumulate both SA and gentisic acid following pathogen challenge, tobacco plants only accumulate SA. Additionally, exogenous SA has a longer half-life in tobacco plants than in tomato plants, which quickly convert exogenous SA to gentisic acid (30). Because SA has a longer half-life in tobacco than in tomato, we hypothesized that even though SA degradation did not detectably contribute to *R. solanacearum* on tomato, it would play a role in tobacco. *R. solanacearum* strain GMI1000 is not a tobacco pathogen because this strain expresses two type III secretion effectors (RipAA and RipP1) that trigger an incompatible hypersensitive response in tobacco (9). We therefore used the tobacco-pathogenic strain K60 to test the contribution of SA degradation to virulence on tobacco. Because strain K60 naturally lacks the SA degradation gene cluster (Fig. 1B), we ectopically expressed the GMI1000 *nag* genes in this strain (Fig. 2A). K60 expressing the *nagAaGHAbIKL*_GMI1000_ gene cluster alone did not grow on SA (Fig. 6A). Because our functional genomics analysis (Fig. 1B) suggests that the *pcaK*_GMI1000_ (porin), RSc1092_GMI1000_ (regulator), and RSc1084_GMI1000_ (porin) genes may also be required for the SA degradation phenotype, we constructed strain K60+KRNP, which expressed 10 genes apparently required for SA degradation: *pcaK*_GMI1000_, RSc1092_GMI1000_, *nagAaGHAbIKL*_GMI1000_, and RSc1084_GMI1000_. Adding these genes allowed K60 to grow on SA as a sole carbon source (Fig. 6A). The wild-type strain K60 was more susceptible to SA toxicity than strain GMI1000, but ectopic expression of the *nag* genes moderately protected strain K60+KRNP from SA toxicity. However, the gene cluster did not confer GMI1000-level SA tolerance on K60 (Fig. 4C).

Acquiring SA degradation capacity increased the virulence of strain K60 on tobacco when bacteria were inoculated into the soil of unwounded tobacco plants (Fig. 6B). The SA-degrading strain (K60+KRNP) also trended toward higher bacterial populations in tobacco stems following soil soak inoculation (Fig. 6C) and trended toward higher populations in tobacco leaf apoplast following leaf infiltration (see Fig. S2A in the supplemental material). However, SA degradation by K60+KRNP did not affect leaf expression of the tobacco defense gene *PR1*, which is activated by SA signaling (see Fig. S2B). The wild-type and SA-degrading strains induced *PR1* expression similarly, relative to mock-inoculated plants, at 10 h postinfiltration with 5 × 10^7^ CFU/ml (see Fig. S2B) and 24 h postinfiltration with 1 × 10^5^ CFU/ml (data not shown). The SA-degrading K60+KRNP strain had wild-type levels of virulence on tomato (Fig. 5D).

## DISCUSSION

SA plays dual roles in plant defense: it is both an antimicrobial compound and a plant defense signaling molecule. Our experiments tested the hypotheses that the plant pathogen *R. solanacearum* uses its Nag degradation pathway to overcome SA toxicity and/or to manipulate host signal transduction. An *R. solanacearum* mutant lacking the Nag pathway was an order of magnitude more sensitive to SA *in vitro*, unambiguously showing that SA degradation protects the bacterium from SA toxicity. However, under our conditions the *nag* genes did not contribute to the virulence on tomato of two *R. solanacearum* strains, GMI1000 and K60. This may be because in tomato, gentisic acid is a more important signaling molecule than SA (30). Consistent with this idea, gentisic acid was less toxic to *R. solanacearum* than SA, and gentisic acid did not induce expression of the *nag* genes in culture. Furthermore, a tobacco-pathogenic *R. solanacearum* strain, K60, became more virulent when it ectopically expressed the *nag* cluster from strain GMI1000. This could be explained by the greater role SA plays in defense signaling in tobacco than for tomato, where gentisic acid works independently from SA (30, 46). It could also be because tobacco plants infected by *R. solanacearum* may contain enough SA to inhibit *R. solanacearum* growth, while tomato plants do not.

Measuring SA concentrations *in planta* is complicated by the multiple bioactive chemical forms of the compound and by the variable spatial and temporal distributions of free SA and its various conjugates (23, 47, 48). However, it is known that free SA can accumulate in plant tissue to levels that inhibit *R. solanacearum* growth; for example, phloem sap of cucumber plants undergoing systemic acquired resistance contains more than 600 µM SA (40). Similarly, apoplastic fluid from *Arabidopsis thaliana* leaves responding to *Pseudomonas syringae* inhibited bacterial growth in an SA-dependent manner (34). A bacterial biosensor designed to measure free SA concentrations *in situ* detected more than 380 µM SA in tobacco leaves responding to tobacco mosaic virus (TMV); this finding corresponds to the infiltrated tobacco leaves in which we compared growth of the wild-type and SA-degrading variant of strain K60 (49). There are no measurements of SA concentrations in plant tissue during bacterial wilt disease, and SA levels in xylem sap have not been well investigated. A liquid chromatography-mass spectrometry analysis determined that SA is present at 3 µM in bulk xylem sap from canola plants infected with *Verticillium longisporum*, while SA was undetected in xylem sap from healthy plants (50). At 3 µM, SA could serve as a carbon source for *R. solanacearum*, but this concentration would not inhibit *R. solanacearum* growth. However, direct chemical analysis of bulk xylem sap to determine if SA accumulates to inhibitory levels cannot take into account microenvironments where SA might reach higher concentrations. *In situ* SA quantification showed that SA concentrations varied 100-fold across tobacco leaves infected with TMV or *P. syringae*, showing that microenvironments within the plant can contain different amounts of SA (49). Although harvested xylem sap would indicate mean SA levels experienced by individual bacteria living as planktonic cells in xylem vessels, it would not measure the SA levels experienced by the large populations of *R. solanacearum* cells that live in biofilm-like aggregates on the xylem wall and in the surrounding tissue (51; D. Khokani and C. Allen, unpublished data). It would be interesting to develop a biosensor strain of *R. solanacearum* that could report the levels of free SA experienced by the pathogen *in situ* during disease development.

The global transcriptional response of *R. solanacearum* to 500 µM SA indicated that exposure to this chemical is stressful to the pathogen, consistent with our observation that a higher SA concentration (600 µM) inhibits growth of *R. solanacearum*. SA repressed diverse genes encoding ribosomal and translational machinery, and SA induced expression of oxidative stress genes, mobile genetic elements, and drug efflux pump genes. Similarly, *Bacillus subtilis* also downregulates ribosomal and translation machinery genes in response to a subinhibitory concentration of 4 mM SA (33). SA induces drug efflux pump gene expression in many bacteria, including non-plant pathogens (32); these pumps increase nonspecific resistance against antibiotics and other toxic chemicals. We previously observed that the DinF and AcrA drug efflux pumps of *R. solanacearum* strain K60 were not responsive to or protective against SA, although these pumps contributed to virulence (31). Our transcriptional profiling revealed that a different drug efflux pump in the MFS family (encoded by RSp0440-2) was highly induced by SA in strain GMI1000, which suggests that this pump may efflux SA from the bacterial cell. Measuring the SA tolerance of a mutant lacking RSp0440-2 could test this hypothesis.

Many pathogens have evolved ways to manipulate SA and SA signaling in order to suppress plant defenses (52). For example, several effectors and toxins take advantage of the natural antagonism between jasmonic acid and SA signaling. The *P. syringae* phytotoxin coronitine, the *P. syringae* effectors HopZ1a and HopX1, and the *Hyaloperonospora Arabidopsis* effector HaRxL44 all induce jasmonic acid signaling to the detriment of SA signaling (53–56). Additional effectors, such as *R. solanacearum* RipR (formerly PopS) and other AvrE family effectors, suppress SA-triggered defenses through unknown mechanisms (21, 52, 57, 58). Plant pathogens also directly manipulate levels of SA; for example *P. syringae* uses the effector HopI1 to limit SA accumulation in the chloroplast (59). The pathogen effectors *Phytophthora sojae* PsIsc1, *Verticillium dahliae* VdIsc1, and *Ustilago maydis* Cmu1 all degrade precursors of SA and thereby limit the host’s ability to synthesize SA (60, 61). *U. maydis* can also directly degrade SA to catechol via Shy1, but Shy1 does not contribute to virulence on maize (62). In contrast, we found that *R. solanacearum*’s ability to degrade SA did not affect expression of the SA marker gene *PR1* in tobacco leaves. To assess responses of plant tissue that was uniformly exposed to the bacterium for the same amount of time, we measured defense gene expression in tobacco leaves following infusion with a bacterial suspension. It is possible that SA degradation does affect defense gene expression in vascular tissue, which is the natural habitat of this pathogen, but our data do not indicate that *R. solanacearum* SA degradation manipulates plant defense gene expression.

Following exposure to 500 µM SA, *R. solanacearum* cells displayed reduced expression of virulence genes encoding type III secretion components, type 4 pilus proteins, extracellular polysaccharide biosynthesis enzymes, and cell wall-degrading enzymes. This is consistent with multiple observations that SA represses virulence genes in bacterial pathogens of plants and animals (32). For example, type III secretion genes are affected by SA or related phenolic compounds in *Erwinia amylovora*, *Dickeya dadantii*, and *Pseudomonas aeruginosa* (63–65). SA also represses biofilm formation and toxin production in *P. aeruginosa*, capsular polysaccharide production by *Klebsiella pneumoniae*, and fimbriae in *Escherichia coli* (66–68). One possible interpretation of the *R. solanacearum* transcriptomic response observed in this study is that SA directly downregulates these virulence genes. The interpretation that we favor, however, is that virulence factors were downregulated by the bacterium in response to the toxicity of SA. Consistent with this interpretation, our qRT-PCR analysis showed that 500 µM SA, but not 50 or 5 µM SA, reduced expression of the type III secretion system effector *ripX*. Taken as a whole, the transcriptomic profile indicates that when *R. solanacearum* is exposed to high, but subinhibitory, concentrations of SA, it pivots from a virulence strategy to a survival strategy. This interpretation is supported by our previous *in planta* transcriptomic analysis that documented 3- to 7-fold-increased expression of the *nag* genes when *R. solanacearum* GMI1000 infected tomato plants at a suboptimally cool temperature compared to at the bacterium’s preferred tropical temperature (29). This suggests that expression of SA degradation genes may be tied to stressful environmental conditions.

The *R. solanacearum* nag cluster includes genes encoding two predicted porins, a predicted LysR transcriptional regulator, and seven SA degradation enzymes. Transcriptional and growth analyses demonstrated that expression of these genes was induced by SA but not by the SA degradation intermediate and plant defense hormone gentisic acid. Ectopic expression studies demonstrated that the seven enzymes are insufficient to confer the ability to grow on SA as a sole carbon source, but expression of the *nag* genes, both porins, and the regulator enabled growth on SA. This functional result suggests that the porins are involved in SA uptake and that some or all of these functions are controlled by the LysR regulator of previously unknown function. However, the genes encoding the porins and regulator should be individually mutated to confirm that *R. solanacearum* requires these genes to grow on SA.

The *nag* cluster is widespread, but not universally conserved, in the *R. solanacearum* species complex. The distribution of this cluster might suggest that this SA degradation contributes to this organism’s fitness. However, an alternate interpretation is that this trait has undergone decay, as suggested by the pattern of loss of SA degradation ability in phylotype IIA and phylotype IV strains. The absence of an SA degradation ability does not correlate with the host range or geographical origin of the strains. It is possible that phylotype IIA and IV strains have diverged in their niche or acquired a trait redundant with SA degradation. *R. solanacearum* strains infect plant hosts from over 250 species, but little is known about the SA concentration and function beyond a few model plants. It is likely that the ability to degrade SA contributes variably to the fitness of *R. solanacearum* species complex strains, depending on the host plant, which ranges from monocots like banana and ginger to dicots like peanut, eucalyptus, and solanaceous plants (20). Because most *R. solanacearum* strains share tomato plants as a host, tomato has been used as an economically important and agriculturally relevant model system to investigate bacterial wilt virulence factors. Our findings offer a reminder that virulence traits may be host specific and emphasize the importance of investigating virulence defects on multiple host plants.

## MATERIALS AND METHODS

### Bacterial culture and growth.

*Escherichia coli* was grown at 37°C in LB. *R. solanacearum* was routinely cultured in CPG rich medium at 28°C (69). When appropriate, antibiotics were added to final concentrations of 25 mg/liter kanamycin, 25 mg/liter gentamicin, and 20 mg/liter spectinomycin. To determine carbon source utilization phenotypes, strains were grown in Boucher’s minimal medium (BMM; pH 7.0) without supplemental carbon as a negative control, with 200 µM sodium succinate as a positive control, or with 200 µM sodium salicylate (70). A plate assay was used to screen *R. solanacearum* isolates for growth on SA. Strains were grown overnight in CPG, and 2-µl aliquots of the cultures were spotted onto BMM agar plates without carbon or supplemented with 1 mM sodium succinate or with 1 mM SA. Growth of each strain was monitored for up to 1 week. For the growth inhibition assays, *R. solanacearum* was grown in BMM pH 5.5 with 10 mM succinate as a carbon source and the indicated concentrations of SA and gentisic acid. Growth was measured using a Synergy HT plate reader (BioTek, Winooski, VT).

### Strain construction.

Bacterial strains, plasmids, and primers are described in Table S2 in the supplemental material. The unmarked Δ*nagGH* mutant was created using a derivative of the *sacB* positive selection vector pUFR80 (71). The primers nagGupF and nagGupR amplified 983 bp upstream of *nagG*, and the primers nagHdwnF and nagHdwnR amplified 1,021 bp downstream of *nagH*. These regions were subcloned into pCR-Blunt (Life Technologies, Grand Island, NY). A HindIII- and SacI-digested fragment was ligated into pUFR80 to create pUFR80-KOnagGH. This plasmid was electroporated into *R. solanacearum* strain GMI1000, and clones with the plasmid integrated into their chromosome were selected on kanamycin plates. Kan^r^ transformants were counterselected on CPG with 5% (wt/vol) sucrose, which selects for a homologous recombination event that results in the loss of the *sacB* gene that confers sucrose susceptibility. The resulting transformants were PCR screened to determine whether they had the wild-type genotype or the *nagGH* deletion.

The Δ*nagAaGHAbIKL* mutant was created by replacing the *nagAaGHAbIKL* region with the spectinomycin resistance cassette from pCR8 (Life Technologies, Grand Island, NY). Specifically, Gibson assembly was used to create the knockout vector pST-KOnagAaGHAbIKL (72). The primers nagAaUpF and nagAaUpR amplified 525 bp upstream of *nagAa*, SmR(nag)F and SmR(nag)R amplified the omega cassette from pCR8, and nagLdwnF and nagLdwnR amplified the 831 bp downstream of *nagL*. Each of these primers has a 5′ sequence that overlaps the neighboring fragment; the overlaps were designed using the NEBuilder software (version 1.10.5; New England Biolabs). The knockout construct was assembled into the HindIII site of pST-Blue. Correct assembly was determined by diagnostic restriction digestions and sequencing. Finally, pST-KOnagAaGHAbIKL was linearized by ScaI digestion and electroporated into GMI1000. Transformants were selected on spectinomycin, and the genotype was verified by PCR screening.

The Δ*nagGH* and Δ*nagAaGHAbIKL* mutants were complemented by inserting the genes with their predicted native promoters into the *R. solanacearum* chromosome at the selectively neutral *att* site. To complement the Δ*nagGH* strain, a 3.3-kb region encompassing the putative promoter, *nagAa*_GMI1000_, *nagG*_GMI1000_, and *nagH*_GMI1000_, was amplified with the primers nagGHCompF and nagGHCompR, subcloned into pCR-blunt, and moved to pRCG-GWY (73) with KpnI and SalI digestion and ligation. The resulting vector, pRCGnagGHcomp, was linearized with ScaI and electroporated into the Δ*nagGH* mutant; transformants were selected on gentamicin plates. To complement the Δ*nagAaGHAbIKL* mutant, a 6.1-kb region encompassing the putative promoter and *nagAaGHAbIKL*_GMI1000_ were amplified using primers nagAaGHAbIKL-F and nagAaGHAbIKL-R and ligated into the HindIII cut site of pUC18miniTn7t-Gm (74) by using Gibson assembly, yielding pMiniTn7-nagAaGHAbIKL-comp. The pMiniTn7-nagAaGHAbIKL-comp vector and the transposase-encoding helper vector pTNS1 were electroporated into the Δ*nagAaGHAbIKL* mutant. Gentamicin-resistant transformants were screened by PCR to confirm the expected incorporation of the vectors into the chromosome. The complemented strains were phenotypically validated by determining that they grew on SA as a sole carbon source.

SA degradation ability was ectopically added to *R. solanacearum* strain K60. Plasmid pMiniTn7-KRNP was created by amplifying the 8.4-kb region encompassing *pcaK*_GMI1000_, RSc1092_GMI1000_, *nagAaGHAbIKL*_GMI1000_, and RSc1084_GMI1000_ with the primers pcaK-F and RSc1084-R and assembling it into pUC18miniTn7t-Gm at the HindIII site. Strain K60 was electroporated with pTNS1 and pMiniTn7-nagAaGHAbIKL-comp or pMiniTn7-KRNP to yield K60+N (expressing *nagAaGHAbIKL*_GMI1000_) or K60+KRNP (expressing *nagAaGHAbIKL*_GMI1000_ plus the putative regulator and two porins), respectively. Strains were PCR screened to confirm the expected genotype.

### Plant growth conditions and inoculations.

Tobacco (cultivar Bottom Special) and tomato (cultivar Bonny Best) plants were grown in 28°C growth chambers with a 16-h day/8-h night cycle. The naturalistic soil soak virulence assay was previously described (75). Briefly, unwounded 17-day-old tomato plants or unwounded 3- to 4-week-old tobacco plants were inoculated by pouring 50 ml of bacterial suspension into the soil to a final concentration of 10^8^ CFU/g soil. Symptoms of each plant were scored daily, using a disease index scale from 0 to 4, corresponding to 0%, <25%, <50%, <75%, or up to 100% leaves wilted, respectively. Virulence was also assessed using the same disease index scale following direct inoculation of 21-day-old tomato plants by placing 500 CFU bacteria on a freshly cut leaf petiole. For tobacco leaf apoplast colonization experiments, fully expanded leaves were syringe infiltrated with a 1 × 10^5^-CFU/ml bacterial suspension. To measure tobacco defense gene expression, fully expanded tobacco leaves were infiltrated with a 5 × 10^7^-CFU/ml suspension or water.

To determine bacterial population sizes in plant stems, 100 mg of tissue was destructively harvested at the cotyledons (for tomato) or at the base of the stem (for tobacco). To sample leaf tissue for colonization, a cork borer was used to excise 1 cm^2^ leaf tissue (approximately 33 mg). The tissue was ground in water in a Powerlyzer bead-beating grinder (MoBio, Carlsbad, CA) and dilution plated to quantify CFU.

### Transcriptional analysis.

The transcriptomic response of *R. solanacearum* to SA was measured using a previously described and validated custom-designed Roche Nimblegen microarray chip with 60,608 gene-specific probes representing 5,061 out of 5,206 open reading frames (ORFs) and 9,549 probes covering the 2,213 unique intergenic regions at 50-bp intervals (28). Strain GMI1000 was grown overnight as three biological replicates. Cells were resuspended in 30 ml of BMM pH 7.0 with 10 mM sodium succinate with or without 500 µM sodium salicylate in a 125-ml flask at a cell density of 1 × 10^8^ CFU/ml. Bacteria were incubated for 3 h to allow cells to acclimate to the conditions before 3.75 ml of RNA stop solution (5% water-saturated phenol in ethanol) was added to the flask. Ten milliliters of cell suspension was centrifuged at 8,000 rpm for 5 min, and pellets were frozen in liquid nitrogen. RNA extraction, DNase treatments, cDNA synthesis, labeling, hybridization and microarray data processing, and statistical analysis were performed as described elsewhere (28). PCR with the universal 759/760 *R. solanacearum* primers confirmed complete DNA removal (76). ORFs with relative expression levels greater than 2-fold and an adjusted *P* value of less than 0.05 were classified as differentially expressed.

To validate the microarray results with qRT-PCR, RNA was harvested from bacteria grown in minimal medium with 0, 5, 50, or 500 µM sodium salicylate as described above. Two micrograms of cDNA was synthesized using the SuperScript VILO system and random hexamer oligonucleotides (Life Technologies). After cDNA synthesis, qRT-PCR was performed with 25-µl reaction mixtures using 10 ng cDNA template and Bullseye Evagreen qPCR mastermix according to the manufacturer’s instructions in an Applied Biosystems 7300 real-time PCR system. Gene-specific primers are listed in Table S2 in the supplemental material. All primer sets had tested efficiencies of 90 to 105% and single peaks on the dissociation curve. Relative expression was calculated using the 2^−ΔΔ*CT*^ method, with normalization to expression of *serC* expression. Relative expression values between 0 and 1 were converted to the fold change by calculating the negative inverse value.

For plant RNA analysis, two 1-cm^2^ tissue punches from a single infused leaf were submerged in 900 µl RNA stop solution in a bead beater tube. Four biological replicates were sampled from independent leaves. Plant tissue was immediately ground in a bead-beating grinder (2,200 rpm for 1.5 min). Samples were centrifuged at 14,000 rpm for 7 min at 4°C, the supernatant was removed, and samples were stored at −80°C. RNA extraction and DNase treatments were performed as described previously (28). One microgram of cDNA was synthesized in a 20-µl reaction mixture using SuperScript III reverse transcriptase (Life Technologies) and oligo(dT)_20_ primers according to the manufacturer’s instructions. qRT-PCRs and analysis were set up as described above, except expression of target genes was normalized to that of the tobacco actin gene. cDNA and qRT-PCR reactions without reverse transcriptase were performed to confirm a lack of DNA contamination. Gene-specific primers are listed in Table S2 in the supplemental material).

## SUPPLEMENTAL MATERIAL

Figure S1  *R. solanacearum* strain GMI1000 gene expression in response to SA. RNA was extracted from cells grown in minimal medium with or without SA and analyzed by qRT-PCR. Relative expression levels of *ripX* (A), *nagK* (B), and *nagH* (C) were calculated using the 2^−ΔΔ*CT*^ method with *serC* as an endogenous normalization control gene and 0 µM SA as an experimental control. Error bars indicate standard errors of the means. Asterisks indicate that the mean relative expression was significantly different than the theoretical mean of 1 (*P* < 0.05, one-sample *t* test). Download Figure S1, TIF file, 0.3 MB

Figure S2  Bacterial density and *PR1* expression in tobacco leaves. (A) *R. solanacearum* population size in tobacco leaf apoplast following syringe infiltration with 1 × 10^5^ CFU/ml (*P* > 0.05 at all time points, Mann-Whitney test). (B) Expression of tobacco *PR1* (SA-responsive gene) in leaf tissue 10 h after syringe infiltration with 5 × 10^7^ CFU/ml bacteria or water. Data are presented as the fold change in expression relative to gene expression in water-infiltrated leaves. Expression of *PR1* was normalized to the tobacco *actin* gene (*P* = 0.45, Student’s *t* test). Download Figure S2, TIF file, 0.2 MB

Table S1  Expression patterns of *R. solanacearum* GMI1000 cells exposed to 500 µM SA. Table S1, XLS file, 0.2 MB

Table S2  Strains, plasmids, and primers used in this study. Table S2, DOCX file, 0.03 MB
