# Supplementary figures and images for: Degradation of the Plant Defense Signal Salicylic Acid Protects Ralstonia solanacearum from Toxicity and Enhances Virulence on Tobacco
Source: mBio. 2016 Jun 21;7(3):e00656-16. doi: 10.1128/mBio.00656-16 (PMC4916378; doi:10.1128/mBio.00656-16)

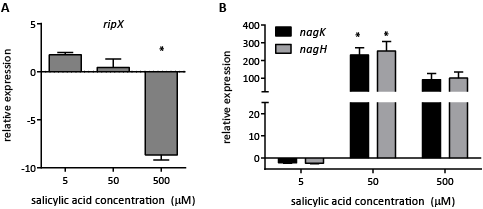

Supplement: Figure S1 — R. solanacearum strain GMI1000 gene expression in response to SA. RNA was extracted from cells grown in minimal medium with or without SA and analyzed by qRT-PCR. Relative expression levels of ripX (A), nagK (B), and nagH (C) were calculated using the 2−ΔΔCT method with serC as an endogenous normalization control gene and 0 µM SA as an experimental control. Error bars indicate standard errors of the means. Asterisks indicate that the mean relative expression was significantly different than the theoretical mean of 1 (P < 0.05, one-sample t test). Download [file mbo003162853sf1.tif]

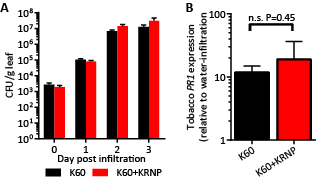

Supplement: Figure S2 — Bacterial density and PR1 expression in tobacco leaves. (A) R. solanacearum population size in tobacco leaf apoplast following syringe infiltration with 1 × 10^5^ CFU/ml (P > 0.05 at all time points, Mann-Whitney test). (B) Expression of tobacco PR1 (SA-responsive gene) in leaf tissue 10 h after syringe infiltration with 5 × 10^7^ CFU/ml bacteria or water. Data are presented as the fold change in expression relative to gene expression in water-infiltrated leaves. Expression of PR1 was normalized to the tobacco actin gene (P = 0.45, Student’s t test). Download [file mbo003162853sf2.tif]
