# Supplementary material for: Degradation of the Plant Defense Signal Salicylic Acid Protects Ralstonia solanacearum from Toxicity and Enhances Virulence on Tobacco
Source: mBio. 2016 Jun 21;7(3):e00656-16. doi: 10.1128/mBio.00656-16 (PMC4916378; doi:10.1128/mBio.00656-16)
Supplement: Table S2 — Strains, plasmids, and primers used in this study. [file mbo003162853st2.docx]

**Table S2**: Strains and plasmids used in this study

| Name | Properties | Reference |
| --- | --- | --- |
| Bacterial strains |  |  |
| *E. coli* |  |  |
| *Top10* | F- mcrA Δ(mrr-hsdRMS-mcrBC) Φ80lacZΔM15 ΔlacX74 recA1 araD139 Δ(ara-leu)7697 galU galK rpsL (Str^r^) endA1 nupG | Life Technologies |
| *R. solanacearum* |  |  |
| GMI1000 | Phylotype I sequevar 18; isolated from tomato, non-pathogenic on tobacco | (1) |
| ΔnagGH | GMI1000 Δ*nagGH* | This study |
| Δ*nagGH* comp | GMI1000 Δ*nagGH* with pRCGnagGHcomp integrated into chromosome; GmR | This study |
| Δ*nagAaGHAbIKL* | GMI1000 Δ*nagAaGHAbIKL*::Ω; Sm^R^ | This study |
| Δ*nagAaGHAbIKL* comp | GMI1000 Δ*nagAaGHAbIKL*::Ω with pMiniTn7-nagAaGHAbIKL-comp integrated into chromosome; Gm^R^ and Sm^R^ | This study |
| K60 | Phylotype IIA; isolated from tomato, pathogenic on tobacco | (2) |
| K60+N | K60 with pMiniTn7-nagAaGHAbIKL-comp integrated into chromosome; Gm^R^ | This study |
| K60+KRNP | K60 with pMiniTn7-KRNP integrated into chromosome; Gm^R^ | This study |
| Grenada91 | Phylotype IIA; isolated from banana | (3) |
| UW181 | Phylotype IIA; isolated from plantain | Allen strain collection |
| CFBP2957 | Phylotype IIA; isolated from tomato | (4) |
| B50 | Phylotype IIA; isolated from banana | (3) |
| IBSBF1900 | Phylotype IIA; isolated from banana | (5) |
| NCPPB282 | Phylotype IIB; isolated from potato | (6) |
| UW551 | Phylotype IIB; isolated from geranium | (7) |
| CFBP1416 | Phylotype IIB; isolated from plantain | (3) |
| CIP417 | Phylotype IIB; isolated from banana | (3) |
| Molk2 | Phylotype IIB; isolated from banana | Boucher, unpublished |
| CFBP6783 | Phylotype IIB; isolated from *Heliconia*, not pathogenic to banana (NPB) | (3) |
| IBSBF1503 | Phylotype IIB; isolated from cucumber, not pathogenic to banana (NPB) | (3) |
| UW163 | Phylotype IIB; isolated from plantain | (3) |
| UW179 | Phylotype IIB; isolated from banana | (3) |
| CMR15 | Phylotype III; isolated from tomato | (4) |
| BDB R229 | Phylotype IV; isolated from banana (host range restricted to banana) | (8) |
| PSI07 | Phylotype IV; isolated from tomato | (4) |
| R. syzygii R24 | Phylotype IV; isolated from clove (host range restricted to clove); insect-transmitted | (8) |
| Plasmids |  |  |
| pUFR80 | Cloning vector for marker-less deletions, Suc^S^ (*sacB*), Km^R^ | (9) |
| pST-Blue | Cloning vector, Km^R^ | Novagen |
| pCR-Blunt | Cloning vector, Km­^R^ | Life Technologies |
| pUC18miniTn7t-Gm | Vector that integrates into selectively neutral *att* site in bacterial chromosomes | (10) |
| pTNS1 | Helper vector for pUC18miniTn7t-Gm, encodes the site-specific TnsABCD transposase, Amp^R^ | (10) |
| pRCG-GWY | Gateway vector that integrates downstream of *glmS* on the GMI1000 chromosome, Gm^R^ | (11) |
| pUFR80-KOnagGH | pUFR80 with 983 bp upstream and 1021 bp downstream of *nagGH* genes fused into MCS, Suc^S^ (*sacB*), Km^R^ | This study |
| pST-KOnagAaGHAbIKL | pST-blue with ΔnagAaGHAbIKL::Sm^R^, Sm^R^, Km^R^ | This study |
| pRCGnagGHcomp | pRCG-GWY with *nagAaGH*_GMI1000_ and the likely native promoter replacing the gateway cassette | This study |
| pMiniTn7-nagAaGHAbIKL-comp | pUC18miniTn7t-Gm with *nagAaGHAbIKL*_GMI1000_ and the likely native promoter | This study |
| pMiniTn7-KRNP | pUC18miniTn7t-Gm with *pcaK*_GMI1000_ porin (K), Rsc1092_GMI1000_ LysR-type regulator (R), *nagAaGHAbIKL*_GMI1000_ (N), RSc1084_GMI1000_ porin (P) and the likely native promoter for *pcaK* | This study |
| Primers |  |  |
| nagGupF | 5`-aaaaagcttcgataacctgctcga  To create pUFR80-KOnagGH | This study |
| nagGupR | 5`-aaatctagacgtttgtctcctgtg  To create pUFR80-KOnagGH | This study |
| nagHdwnF | 5`-aaatctagagacctgccatgacga  To create pUFR80-KOnagGH | This study |
| nagHdwnR | 5`-aaagagctcgttcgtacgggtagc  To create pUFR80-KOnagGH | This study |
| nagAaUpF | 5`-gatatctgaattcgtcgacaaccgtactgaccgtgacgc  To create pST-KOnagAaGHAbIKL | This study |
| nagAaUpR | 5`-atgaccatgtggccagtgtctccgggctg  To create pST-KOnagAaGHAbIKL | This study |
| SmR(nag)F | 5`-gacactggccacatggtcatagctgtttcctgg  To create pST-KOnagAaGHAbIKL | This study |
| SmR(nag)R | 5`-gggctcgccgtaagggattttggtcatgggtgg  To create pST-KOnagAaGHAbIKL | This study |
| nagLdwnF | 5`-aaatcccttacggcgagcccacaaccca  To create pST-KOnagAaGHAbIKL | This study |
| nagLdwnR | 5`-gagctagcctaggctcgagaagtcgtaggcggcggagag  To create pST-KOnagAaGHAbIKL | This study |
| nagGHCompF | 5`-atctcatcgcgcaacgtatc  To create pRCGnagGHcomp | This study |
| nagGHCompR | 5`-atgagcttgaaccaggttgt  To create pRCGnagGHcomp | This study |
| nagAaGHAbIKL-F | 5`-caggaattcctcgagaatctcatcgcgcaacgtatcg  To create pMiniTn7-nagAaGHAbIKL-comp | This study |
| nagAaGHAbIKL-R | 5`-gaggtaccgggcccaactgctgttggacagaccc  To create pMiniTn7-nagAaGHAbIKL-comp | This study |
| pcaK-F | 5`-caggaattcctcgagatctcgcatttgcagcgtc  To create pMiniTn7-KRNP | This study |
| RSc1084-R | 5`-gaggtaccgggcccagacagcttcagcgtctac  To create pMiniTn7-KRNP | This study |
| 759 | 5`-gtcgccgtcaactcactt  Universal *R. solanacearum* primer | (12) |
| 760 | 5`-gtcgccgtcagcaatgcg  Universal *R. solanacearum* primer | (12) |
| popAJMJqGMIF | 5`-TGATCGCCGCCATCGTGCAG  qRT-PCR primer for *ripX* (formerly *popA*) | This study |
| popAJMJqGMIR | 5`-TGTTGCCGATTGCGGACCC  qRT-PCR primer for *ripX* (formerly *popA*) | This study |
| qRT-nagH-F1 | 5`-TTCGAAAGCAAGGGGATGCT  qRT-PCR primer for *nagH* | This study |
| qRT-nagH-R1 | 5`-CTGGTAGTAGGGGTCGTGGA  qRT-PCR primer for *nagH* | This study |
| qRT-nagK-F1 | 5`-GCCGTTCTTCTTCTGCAAGC  qRT-PCR primer for *nagK* | This study |
| qRT-nagK-R1 | 5`-AGTGGTAGTTCTGCGTCTGC  qRT-PCR primer for *nagK* | This study |
| serCFqRTGMI | 5`-CGCGCAAATACGGTGAAGTG  qRT-PCR primer for *serC* | This study |
| serCRqRTGMI | 5`-GTGCACAGATGCACGTAAGC  qRT-PCR primer for *serC* | This study |
| actinU60491F | 5`-ggatcttgctggtcgtga  qRT-PCR primer for *NtActin* | (13) |
| actinU60491R | 5`-cctgcccatctggtaact  qRT-PCR primer for actin | (13) |
| NtPR1-F | 5`-ggatgcccataacacagctc  qRT-PCR primer for *NtPR-1a*, *NtPR-1b*, and *NtPR-1c* | (14) |
| NtPR1-R | 5`-gctaggttttcgccgtattg  qRT-PCR primer for *NtPR-1a*, *NtPR-1b*, and *NtPR-1c* | (14) |

Gm^R^, gentamicin resistance; Km^R^, kanamycin resistance; Sm^R^, spectinomycin resistance; Suc^S^, sucrose sensitivity

**References:**

1. **Salanoubat M, Genin S, Artiguenave F, Gouzy J, Mangenot S, Arlat M, Billault A, Brottier P, Camus JC, Cattolico L, Chandler M, Choisne N, Claudel-Renard C, Cunnac S, Demange N, Gaspin C, Lavie M, Moisan A, Robert C, Saurin W, Schiex T, Siguier P, Thebault P, Whalen M, Wincker P, Levy M, Weissenbach J, Boucher CA.** 2002. Genome sequence of the plant pathogen *Ralstonia solanacearum*. Nature **415:**497-502.

2. **Kelman A.** 1954. The relationship of pathogenicity of *Pseudomonas solanacearum* to colony appearance in tetrazolium medium. Phytopathol **44:**693-695.

3. **Ailloud F, Lowe T, Cellier G, Roche D, Allen C, Prior P.** 2015. Comparative genomic analysis of *Ralstonia solanacearum* reveals candidate genes for host specificity. BMC Genomics **16:**270.

4. **Remenant B, Coupat-Goutaland B, Guidot A, Cellier G, Wicker E, Allen C, Fegan M, Pruvost O, Elbaz M, Calteau A, Salvignol G, Mornico D, Mangenot S, Barbe V, Médigue C, Prior P.** 2010. Genomes of three tomato pathogens within the *Ralstonia solanacearum* species complex reveal significant evolutionary divergence. BMC Genomics **11:**379.

5. **Wicker E, Grassart L, Coranson-Beaudu R, Mian D, Guilbaud C, Fegan M, Prior P.** 2007. *Ralstonia solanacearum* strains from Martinique (French West Indies) exhibiting a new pathogenic potential. Appl Environ Microbiol **73:**6790-6801.

6. **Clarke CR, Studholme DJ, Hayes B, Runde B, Weisberg A, Cai R, Daunay M-c, Wicker E, Castillo JA, Vinatzer BA.** 2015. Genome-enabled phylogeographic investigation of the quarantine pathogen *Ralstonia solanacearum* Race 3 Biovar 2 and screening for sources of resistance against its core effectors. Phytopathol **105:**597-607.

7. **Gabriel DW, Allen C, Schell M, Denny TP, Greenberg JT, Duan YP, Flores-Cruz Z, Huang Q, Clifford JM, Presting G, González ET, Reddy J, Elphinstone J, Swanson J, Yao J, Mulholland V, Liu L, Farmerie W, Patnaikuni M, Balogh B, Norman D, Alvarez A, Castillo JA, Jones J, Saddler G, Walunas T, Zhukov A, Mikhailova N.** 2006. Identification of open reading frames unique to a select agent: *Ralstonia solanacearum* Race 3 biovar 2. Mol Plant Microbe Interact **19:**69-79.

8. **Remenant B, Cambiaire J-CD, Cellier G, Barbe V, Medigue C, Jacobs JM, Fegan M, Allen C, Prior P.** 2011. Phylotype IV strains of *Ralstonia solanacearum, R. syzygii* and the Blood Disease Bacterium form a single genomic species despite their divergent life-styles. PLoS ONE **6:**e24356.

9. **Castañeda A, Reddy JD, El-Yacoubi B, Gabriel DW.** 2005. Mutagenesis of all eight *avr* genes in *Xanthomonas campestris* pv. *campestris* had no detected effect on pathogenicity, but one *avr* gene affected race specificity. Mol Plant-Microbe Interact **18:**1306-1317.

10. **Choi K-H, Gaynor JB, White KG, Lopez C, Bosio CM, Karkhoff-Schweizer RR, Schweizer HP.** 2005. A Tn7-based broad-range bacterial cloning and expression system. Nat Methods **2:**443-448.

11. **Monteiro F, Solé M, Dijk Iv, Valls M.** 2012. A chromosomal insertion toolbox for promoter probing, mutant complementation, and pathogenicity studies in *Ralstonia solanacearum*. Mol Plant Microbe Interact **25:**557-568.

12. **Opina N, Tavner F, Hollway G, Wang J-F, Li T-H, Maghirang R, Fegan M, Hayward AC, Krishnapillai V, Hong WF, Holloway BW, Timmis J.** 1997. A novel method for development of species and strain-specific DNA probes and PCR primers for identifying *Burkholderia solanacearum* (formerly *Pseudomonas solanacearum*). Asia Pacific J Mol Biol Biotech **5:**19-30.

13. **Novák J, Pavlů J, Novák O, Nožková-Hlaváčková V, Špundová M, Hlavinka J, Koukalová Š, Skalák J, Černý M, Brzobohatý B.** 2013. High cytokinin levels induce a hypersensitive-like response in tobacco. Annals of Botany **112:**41-55.

14. **Rivière M-P, Marais A, Ponchet M, Willats W, Galiana E.** 2008. Silencing of acidic pathogenesis-related PR-1 genes increases extracellular β-(1→3)-glucanase activity at the onset of tobacco defence reactions. J Exp Bot **59:**1225-1239.
